# Targeted gene sequencing of *FYCO1* identified a novel mutation in a Pakistani family for autosomal recessive congenital cataract

**DOI:** 10.1002/mgg3.1985

**Published:** 2022-05-31

**Authors:** Rani Saira Saleem, Sorath Noorani Siddiqui, Saba Irshad, Naeem Mahmood Ashraf, Arslan Hamid, Muhammad Azmat Ullah Khan, Muhammad Imran Khan, Shazia Micheal

**Affiliations:** ^1^ School of Biochemistry and Biotechnology University of Punjab Lahore Pakistan; ^2^ Department of Pediatric Ophthalmology and Strabismus Al‐Shifa Eye Trust Hospital Rawalpindi Pakistan; ^3^ Department of Biochemistry and Biotechnology University of Gujrat Punjab Pakistan; ^4^ LIMES Institute University of Bonn Bonn Germany; ^5^ Department of Human Genetics Radboud University Medical Centre Nijmegen The Netherlands; ^6^ Department of Clinical Genetics AcademicMedical Centre Amsterdam The Netherlands

**Keywords:** autophagy, autosomal recessive, congenital cataract, *FYCO1*, lens

## Abstract

**Background:**

Congenital cataract is causing one‐third of blindness worldwide. Congenital cataract is heterogeneous in its inheritance patterns. The current study is aimed to explore the unknown genetic causes underlying congenital cataracts.

**Methods:**

Blood samples from affected and normal individuals of n = 25 Pakistani families identified with congenital cataracts were collected. Genomic DNA was extracted and Sanger sequencing was performed to identify novel pathogenic variants in the *FYCO1* (MIM#607182) gene. Later structural bioinformatics tools and molecular dynamics simulations were performed to analyze the impact of these variants on protein structure and function.

**Results:**

Sanger sequencing resulted in the identification of a novel splice site mutation (NM_024513.3: c.3151‐29_3151‐7del) segregating in an autosomal recessive manner. This novel variant was confirmed to be absent in the n = 300 population controls. Further, bioinformatics tools revealed the formation of a mutant protein with a loss of the Znf domain. In addition, we also found a previously known (c.4127 T > C; p.Leu1376Pro) mutation in four families. We also report a novel heterozygous variant (c.3419G > A; p.Arg1140Gln) in another family.

**Conclusions:**

In conclusion, we report a novel deletion (NM_024513.3: c.3151‐29_3151‐7del) in one family and a frequent homozygous missense mutation (c.4127 T > C; p.Leu1376Pro) in four Pakistani families. The current research highlights the importance of autophagy in lens development and maintaining its transparency.

## Graphical abstract

The current study reports a novel splice site mutation in FYCO1 gene segregating in autosomal recessive manner with Congenital Cataract. The study also predicts the impact of this mutation on protein structure of FYCO1 by using MD simulations. It also emphasize significance of Autophagy in maintaining the lens transparency.

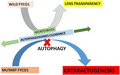

## 1 INTRODUCTION

Congenital cataract (CC) is referred to as the leading cause of reversible blindness in children. Its prevalence is ranging from 2.2 to 13.6 per 10,000 children as given based on population‐based studies worldwide (Wu et al., 2016). The initiation of cataracts occurs mainly when there is an accumulation of proteins in the lens that results in the formation of large aggregates that block the retina's transmission of visible images (Wasnik et al., 2021). The optical properties of the lens depend upon the appropriate cellular architecture of the lens fiber cells and the accumulation of the soluble proteins called crystallins, which maintain a constant refractive index of the lens. Any changes in the structure of the lens result in the opacification of the lens thus, driving the variations of the refractive index of the lens (Shoshany et al., 2020).

Congenital cataract has been classified into different subtypes based upon the genotype–phenotype relationship. These types include nuclear, lamellar, total, cortical, anterior polar, and posterior polar cataracts depending upon the location and morphology of the opacities (Vander Veen, 2012). The clinical and genetic heterogeneity of congenital cataracts makes it much more diverse. Congenital cataract follows all types of inheritance patterns in both syndromic and non‐syndromic, involving about more than 100 causative genes. However, autosomal dominant is the most prevalent mode of inheritance (Hansen et al., 2009).

To date, 1422 disease‐causing variants have been reported for this disease (Wasnik et al., 2021). Thirty‐nine different genes are involved in non‐syndromic inherited congenital cataracts. So far, 14 genetic loci have been implicated in the autosomal recessive congenital cataract (arCC) and a few of the responsible genes are still unknown. Some of the known genes like *CRYBB1* (MIM#600929) (Cohen et al., 2007), *CRYAA* (MIM#123580) (Beby et al., 2007), *CRYBB3* (MIM#123630) (Riazuddin et al., 2005), and *CRYAB* (MIM#123590) (Chen et al., 2009) are involved in both dominant and recessive inheritance patterns whereas, some of the genes like *EPHA2* (MIM#176946) (Kaul et al., 2010)*, BFSP2* (MIM#603212) (Aldahmesh et al., 2011), *HSF4* (MIM#602438) (Behnam et al., 2016)*, CRYBA1* (MIM#123610) (AlFadhli et al., 2012)*, CRYGD* (MIM#123690) (Gao et al., 2016)*, GJA8* (MIM#600897) (Arora et al., 2008; Micheal et al., 2018), *CRYGC* (MIM#123680) (Gonzalez‐Huerta et al., 2007)*, CRYGS* (MIM#123730) (Yang et al., 2013), *VIM* (MIM #193060) (Ma et al., 2016), *MIP* (MIM#154050) (Gu et al., 2007)*, WFS1* (MIM#606201) (Berry et al., 2013), *GJA3* (MIM#121015) (Ding et al., 2011), *TMEM114* (MIM#611579) (Jamieson et al., 2007)*, UNC45B* (MIM#611220) (Hansen et al., 2014)*, PAX6* (MIM#607108) (Brémond‐Gignac et al., 2010), *CHMP4B* (MIM#610897) (Shiels et al., 2007), and *CRYBB2* (MIM#123620) (Faletra et al., 2013) are involved in the autosomal dominant mode of Inheritance only.

The current study is aimed to explore the role of the *FYCO1* (MIM#607182) gene in causing congenital cataracts in Pakistani families with a focus particularly on autophagy in maintaining the transparency of the lens. This autophagy protein FYCO1 expresses itself in newborns and plays a critical role in development of eye lens (Brennan et al., 2012). As compared to other genes, involved in eye disorders, little information is available in literature about variants of *FYCO1* (MIM#607182) in congenital cataracts. Currently, CRISPR/Cas gene therapy techniques are getting lot of attention to cure the genetic diseases, particularly eye disorders (Zhang, 2021). Therefore, there is need to investigate all disease‐causing genetic variants. In this study, we investigated the *FYCO1* gene for novel genetic variants, causing the disease. Furthermore, we have employed molecular dynamics (MD) simulations to understand the impact of the variants on the structure and function of the protein. The resulting data supports the hypothesis that there exists a strong correlation between FYCO1 protein, autophagy, and cataract.

## 2 MATERIALS AND METHODS

### 2.1 Subjects

The recruitment of the families was done from the pediatric ophthalmology department of Al‐Shifa Eye Trust Hospital, Rawalpindi, Pakistan.

### 2.2 Clinical examination

In total, a cohort of n = 25 families was selected for this study with a familial history of congenital cataracts. Detailed ocular, medical and family data were obtained from the available family members of the patients. A complete ophthalmic examination was performed by slit lamp for both affected and non‐affected members of the family.

### 2.3 Blood DNA extraction

Genomic DNA was extracted from whole blood by using the QIAGEN DNA Blood Midi Kit (QIAGEN, Germantown, Maryland, USA) by following the detailed instructions of the given package insert.

### 2.4 Sanger sequencing

The coding regions and the intronic boundaries of the *FYCO1* gene were sequenced in n = 25 probands of the selected families with familial history of congenital cataracts. Gene‐specific primers were designed by using online software Primer 3 to cover all the coding regions of exons and intronic boundaries covering at least 100–120 bp. The PCR product was analyzed with 2% agarose gels. Sanger sequencing was performed by using ABI Big Dye chemistry (Applied Biosystems Inc., Foster City, CA, USA), by using an automated ABI 3730 Sequencer (Applied Biosystems, Inc.) and following the given protocols. Sequence alignment was performed with the reference sequence to find the variant by using CodonCode Aligner (version 6.1) (CodonCode Co., Centerville, MA, USA). The conditions used for PCR reaction mix and annealing temperatures were adapted from the literature. Various online prediction tools like SIFT, MutationTaster, and PolyPhen‐2 were used to determine the pathogenicity of missense variants. For splice site variants, MaxEntScan, NNSPLICE, GeneSplicer, Human Splicing Finder, and ESE were used for splicing predictions.

### 2.5 Bioinformatics analysis

The three‐dimensional prediction of protein structure was done by using the online server I‐TASSER (Yang et al., 2015). This server used a threading algorithm to predict the structure of the protein. I‐TASSER predicted five potential models. Among these, the best model was selected based on the highest C‐score. InterPro server was used to identify the conserved domains and their functions in the protein structure (Blum et al., 2021). Finally, 20 ns molecular dynamics (MD) simulations were performed using the GROMACS 5.0.5 tool to observe the dynamic impact of mutations on the protein structure (Van Der Spoel et al., 2005). Root mean square deviation (RMSD), root mean square fluctuations (RMSF), radius of gyration (Rg), and the total number of hydrogen bonds (Hn) were analyzed by using trajectory.

## 3 RESULTS

### 3.1 Family 1 with novel mutation (NM_024513.3: C.3151‐29_3151‐7del)

**FIGURE 1:**
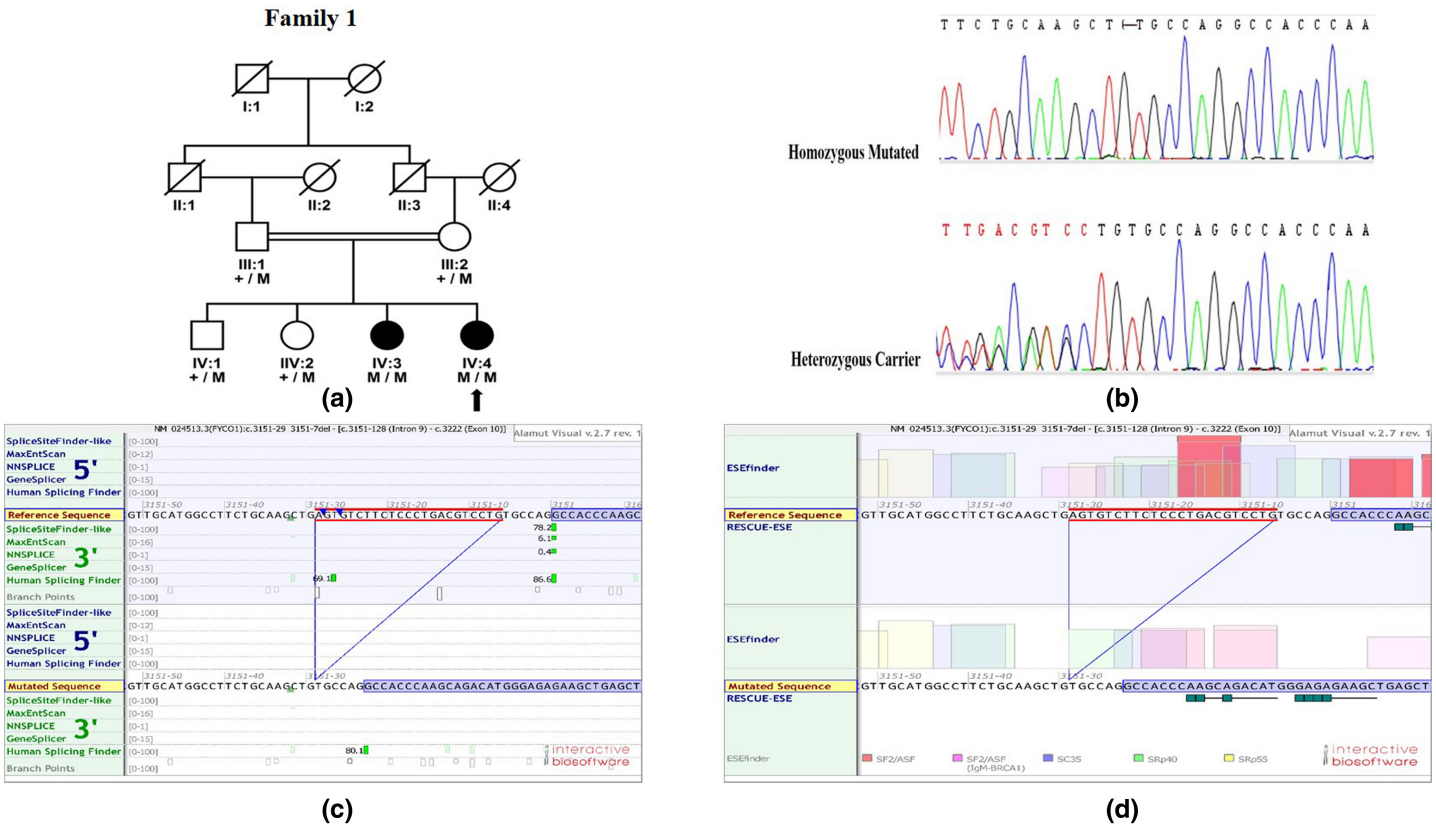
Novel mutation (NM_024513.3: C.3151‐29_3151‐7del). (a) A four‐generation consanguineous family with inherited congenital cataract. The proband is shown with an arrow. (b) DNA Sequence Chromatogram analysis with the sequence of affected homozygous mutated individual with a heterozygous carrier individual. (c) Ex‐SKIP and Splice Site predictor Score. (d) ESE Predictions

In family 1, there were two affected and four unaffected members as shown in the pedigree (Figure 1a). The proband (IV: 4) was a 6‐year‐old girl. Both affected members have nuclear cataracts with no other ocular or systemic abnormalities. Sanger Sequencing resulted in the identification of a novel mutation (NM_024513.3^1^: c.3151‐29_3151‐7del) in the intronic region. This novel deletion was found to be segregating with the disease homozygously among the affected and unaffected members of the family (Figure 1b). This novel mutation (NM_024513.3: c.3151‐29_3151‐7del) is expected to result in the disruption of the splice site of exon 10 with a probable consequence of skipping exon 10 during transcription. The calculations for the Ex‐SKIPscore and Splice Site predictor present that this change leads to the possibility of a loss of splice site of exon 10 as shown in Figure 1c,d. As a result, the mRNA formed will be shorter in length by 119 bp with a loss of about 41 amino acid residues from 1051 to 1090 as compared to wild type RefSeq NP_078789. This novel variant was verified to be absent in population controls of about n = 300 and the ClinVar database.

**FIGURE 2:**
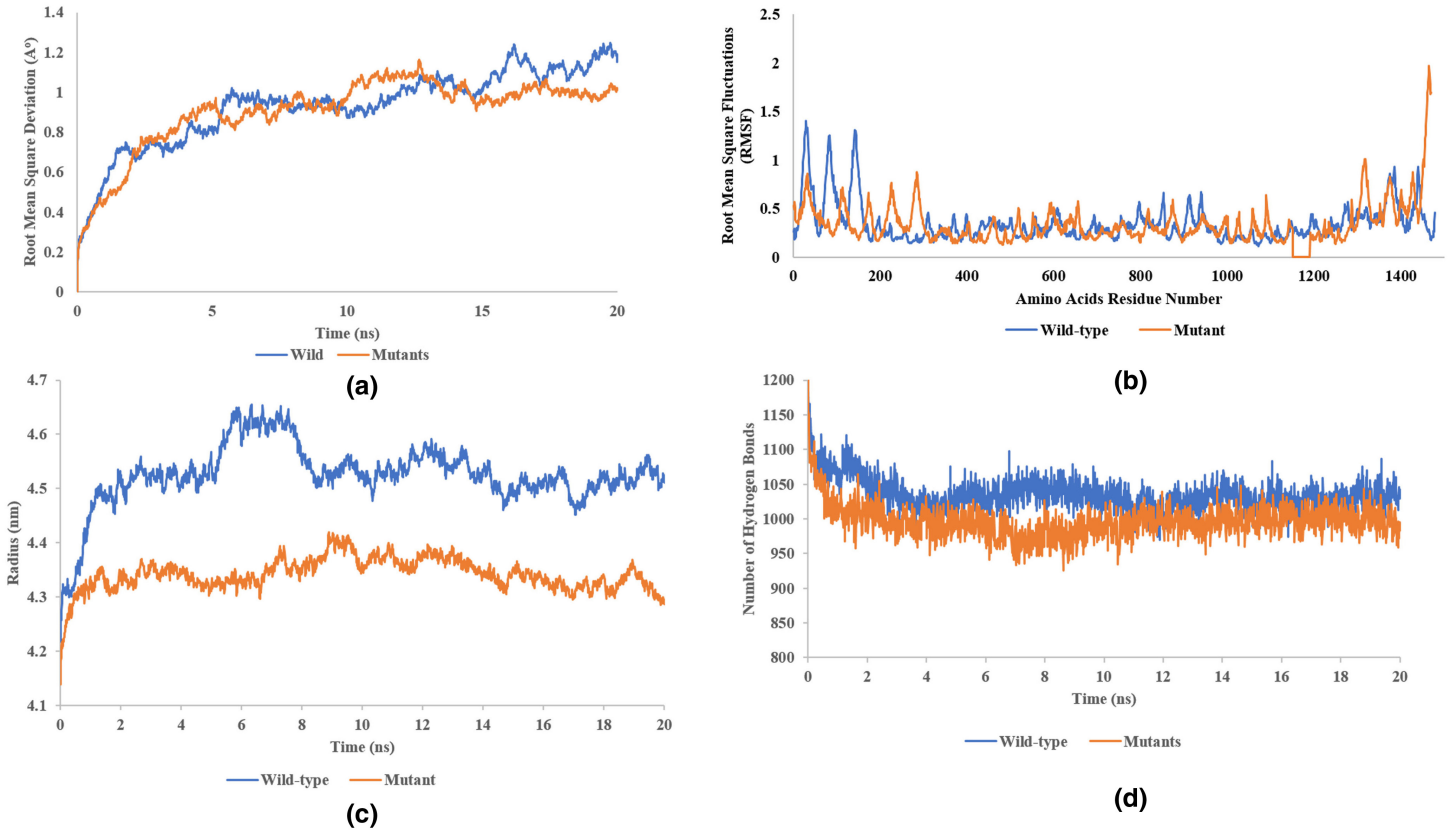
Bioinformatics analysis of novel mutation (NM_024513.3: C.3151‐29_3151‐7del). (a) RMSD graph of wild‐type and mutant structures of Molecular Dynamics trajectory. The wild‐type and mutants are showing similar trends, indicating that mutation does not impact the overall 3D structure of the protein. (b) RMSF graph of wild‐type and mutants structures amino acids residues. These values are calculated during 20 ns Molecular Dynamics simulations. There are significant differences of fluctuations between some wild‐type and mutant parts. (c) Radius of Gyration (Rg) of wild‐type and mutant calculated during 20 ns Molecular Dynamics simulations. There is a significant difference between Rg value of wild type and mutant structures. (d) Total number of hydrogen bonds calculated in wild‐type and mutant structures during 20 ns Molecular Dynamics simulations. Almost identical numbers of hydrogen bonds are observed in wild‐type and mutants

We analyzed the dynamic impact of this mutation on the structure of the protein by using a 20 ns molecular dynamics simulation. GROMACS 5.0.5 version was used for this purpose. Root mean square deviation (RMSD), root mean square fluctuation (RMSF), the radius of gyration (Rg), and the total number of hydrogen bonds (Hn) were calculated for wild‐type and the mutant structure. It was found that both wild‐type and the mutated structures showed a similar type of RMSD trend (Figure 2a). This indicates that the mutation does not disturb the overall structure of the protein. However, in RMSF, fewer fluctuations were observed in the mutant structure as compared to the wild‐type. The Rg value for the mutant structure was calculated to be 4.3 which was lesser than the Rg value of the wild‐type that is, 4.6. From this RMSF and Rg data, it is concluded that the mutant protein structure as a result of this mutation became more compact as compared to the wild‐type (Figure 2b,c). However, almost same number of hydrogen bonds were seen in 20 ns simulations (Figure 2d). This shows that due to this mutation the mutant protein structure failed to adopt necessary conformational changes thereby failing to carry out its role in signaling cascades of autophagy.

### 3.2 Mutation (c.4127 T > C: P.Leu1376Pro)

**FIGURE 3:**
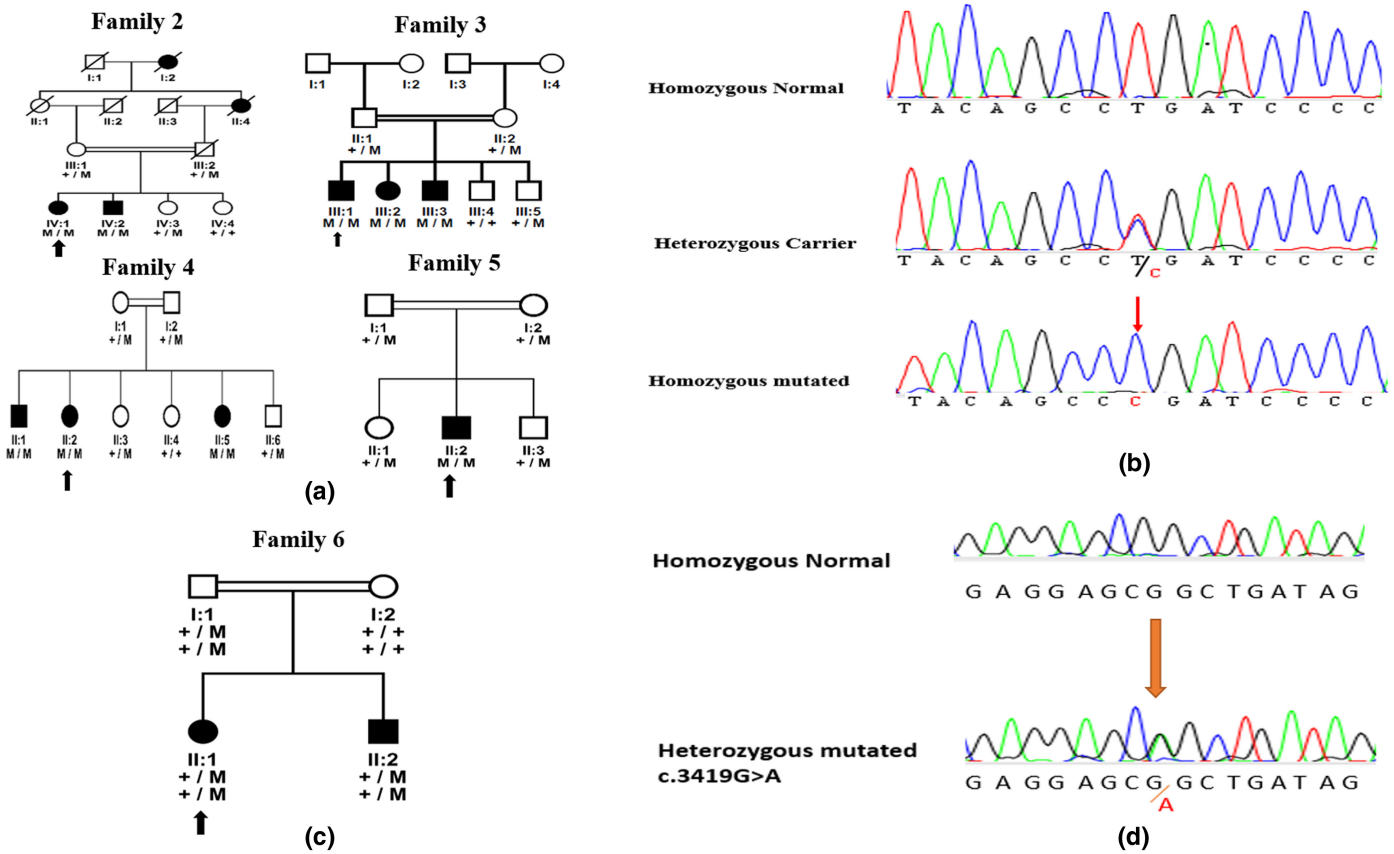
Mutation (c.4127 T > C; p.Leu1376Pro) and heterozygous variant (c.3419G > a; p.Arg1140Gln). (a) Pedigree of families with inherited congenital cataract (2‐5). (b) Mutation analysis of homozygous mutated individual along with the heterozygous carrier and normal individual for already reported mutation (c.4127 T > C; p.Leu1376Pro). (c) Pedigree of a family 6 showing heterozygous variant (c.3419G > A; p.Arg1140Gln). (d) Mutation analysis of homozygous normal individual along with the heterozygous mutated individual for variant (c.3419G > A; p.Arg1140Gln)

A previously known mutation (c.4127 T > C; p.Leu1376Pro) was also found in four families 2, 3, 4 & 5 as shown in Figure 3a, of our cohort. This mutation (c.4127 T > C; p.Leu1376Pro) was found to be segregating with the affected and unaffected individuals of these families (Figure 3b). This mutation leads to the loss of highly conserved Leu1376 of mammalian species. This reported missense change actually disrupts the reading frame for the GOLD domain of FYVE Coiled‐coil protein which is very critical for the normal functioning of the protein (Chen et al., 2011). The presence of this mutation in four out of 25 families suggests it to be a frequent mutation of *FYCO1* in the cases of congenital cataracts of Pakistani origin. The change (NP_078789: p.Leu1376Pro) has already been reported to be possibly damaging by PolyPhen and deleterious by SIFT.

### 3.3 Family 6 with heterozygous variant (c.3419G > a; p.Arg1140Gln)

We report another novel heterozygous variant in family 6, with a likely benign status (c.3419G > A; p.Arg1140Gln) as shown in Figure 3c,d. This variant has a disease‐causing with *p*‐value = 1 by mutation transfer due to change in highly conserved nucleotide thus giving Polyphen score: 5.94. However, the variant failed to segregate with the affected and unaffected individuals of the family thus indicating that this might not be the only cause of congenital cataract in this family.

In addition, there are some other common Synonymous and non‐synonymous variants found in these families for *FYCO1* that have been listed in Tables 1 and 2, respectively.

**TABLE 1:** Frequency of synonymous/noncoding variants in Pakistani Families

Exon	Variant SNP ref as;	cDNA position	Amino acid change:	Homo/Het	Clinical sig:	Frequency out of 25	%age
E_4	rs4682801	c.267C > A	(p.Arg89Arg)	Hom/Het	Benign	21	84%
E_8–1	rs13071283	c.819A > G	p.Gln273Gln	Het	Benign	10	40%
E_8–2	rs3796376	c.1335G > A	p.Leu445Leu	Hom/Het	Benign	14	56%
E_8–5	rs13079869	c.2739C > T	p.Cys913Cys	Hom/Het	Benign	9	36%
E_13	rs41289618	c.3789A > G	p.Thr1263Thr	Het	Likely benign	1	4%
E_14	rs1463680	c.3924C > T	p.Leu1308Leu	Hom/Het	Benign	24	96%
E_5	rs751552	c.289‐14 T > A	‐	Het	Benign	16	64%
E_6	rs41289622	c.539 + 35A > C	‐	Het	‐	6	24%
E_12	rs13069079 rs1532071	c.3587 + 15C > T c.3438–29C > T	‐	Het Hom/Het	Benign	11 15	44% 60%

*Note*: Nucleotide and amino acid designations are based on Refseq (NM_024513.3) and (NP_078789). An updated version of (NM_024513.3) may be found as (NM_024513.4).

**TABLE 2:** Frequency of non‐synonymous variants in Pakistani families

Exon	Variant SNP ref as;	cDNA position	Amino acid change:	Homo/Het	Clinical sig:	Frequency out of 25	%age
E_8–1	rs4683158 rs3733100	c.749G > A c.962G > C	p.Arg250Gln p.Gly321Ala	Hom Hom/Het	Benign Benign	22 14	85% 56%
E_8–2	rs33910087	c.1339C > T	p.Arg447Cys	Hom/Het	Benign	9	36%
E_8–3	rs3796375	c.2036C > T	p.Ala679Val	Hom/Het	Benign	9	36%
E_8–5	rs13059238 rs13079478	c.3001A > G c.3003C > A	p.Asn1001Asp p.Asn1001Lys	Hom/Het Hom/Het	Benign Benign	9 8	36% 32%
E_11	rs41289620	c.3419G > A	p.Arg1140Gln	Het	Benign. likely benign	1	4%
E_16	rs387906965	c.4127 T > C	p.Leu1376Pro	Hom	pathogenic	4	16%

## 4 DISCUSSION

**FIGURE 4:**
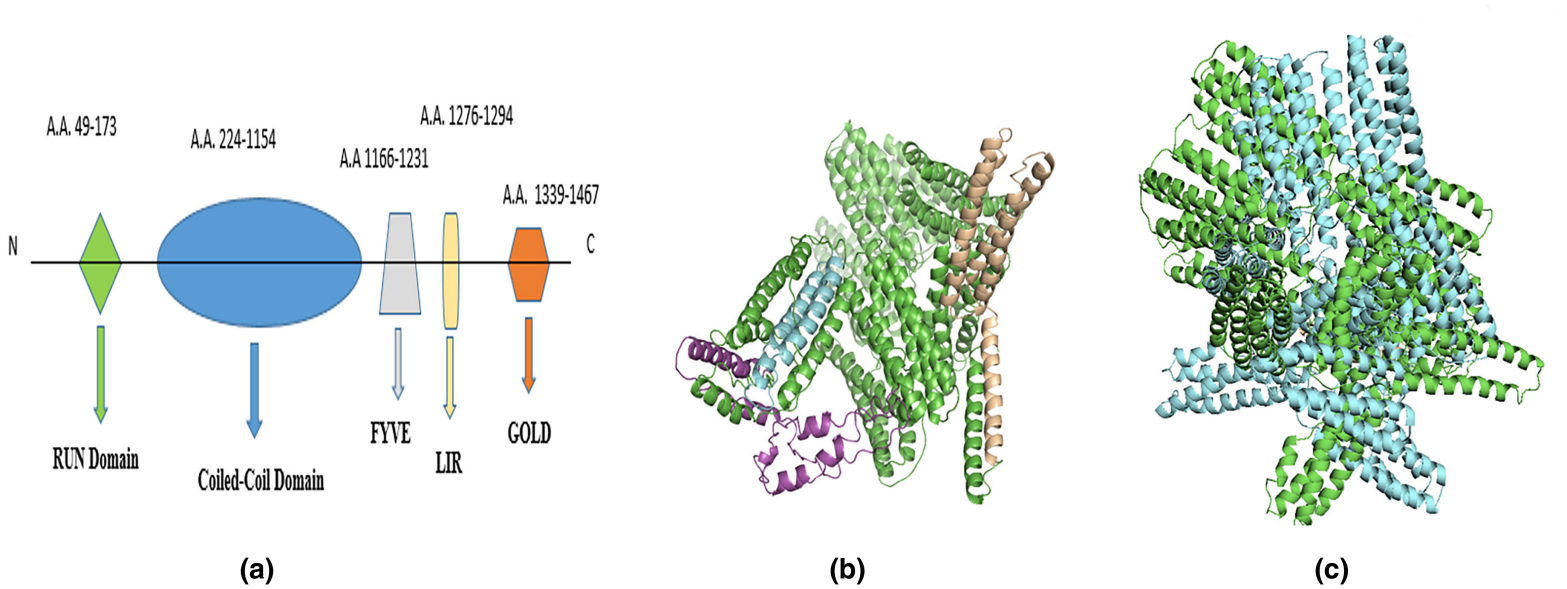
InterPro and 3D structural alignment of FYCO1 protein domains. (4a) Domain Structure for FYVE coiled‐coil Protein. (b) 3D structure of wild‐type protein, including Run Domain (Gold‐Wheat), Znf Domain (Cyan), and GOLD domain (Magenta). (c) Structural alignment of wild‐type and mutant structure. Wild‐type and mutant structures showed poor structural alignment. This indicates that mutation resulted in a conformational changes in the protein structure

In our study, we identified a novel mutation (NM_024513.3: c.3151‐29_3151‐7del) in the flanking region of Exon 10 thus disrupting splice site for post‐transcriptional modifications. The gene for FYVE and coiled‐coil domain containing 1 (*FYCO1*) is located on chromosome 3 at position 3p21.31. This gene is comprised of 18 exons and exon 1 is non‐coding. The protein coded by this gene consists of 1478 amino acids with a Molecular mass of 166,983 Daltons. InterPro results revealed that there are four domains in the protein, including RUN domain (36–173 aa residues), coiled‐coil domain (224–1154), Znf domain (1166–1231 aa residues), and GOLD domain (1339–1466 aa residues) (Figure 4a).

Out of the 19 mutations reported so far in *FYCO1* for congenital cataract mostly are responsible for the formation of truncated protein hampered with coiled–coil or GOLD domain of the protein (Iqbal et al., 2020). Recently reported mutations in *FYCO1* include c.2365 C > T (Aprahamian et al., 2021), (c.1621C > T; p. Gln541* (Barashkov et al., 2021) and (c.1387 G > T; p.G463X) (Al‐Badran et al., 2022). Among all these, this is the first longest deletion reported so far in *FYCO1* gene and 2nd reported splice site variant for Exon 10 in addition to previously reported splice site variant (c.3150 + 1G > T) (Chen et al., 2011). Exon 10 is involved in the synthesis of the Znf domain which plays an important role in signaling pathways. Previously, only 4 (c.3150 + 1G > T), (c.3151–2A > C; p.A1051DfsX27), (c.3196delC; p.H1066IfsX10), and (c.3670C > T; p.R1224X) mutations have been reported for Znf domain.

In‐silico analysis indicated two major impacts of this mutation on the protein structure. First, it affects the synthesis of the Znf domain of the FYVE and coiled‐coil domain containing 1 (FYCO1) protein, suggesting that this domain is critical for the function of FYCO1 protein. Second, it is the first of the longest known deletion reported so far for *FYCO1*. It is interesting to find that this longest deletion is not leading to the truncation of the protein. However, is leading to loss of the necessary protein conformation as shown by the MD simulations with fewer RMSF fluctuations in the mutant as compared to wild‐type. It means that this novel variation caused to increase overall compactness of the mutant protein structure as shown in Figure 4b,c. These results suggest the importance of the necessary protein confirmation required for FYCO1 to act as an adapter molecule for autophagy. The Znf domain in the protein interacts with DNA, RNA, or proteins. The binding affinities depend upon the sequence of the domain and its proper conformation. In FYCO1, the Znf domain believes to interact with cytoskeleton proteins along with the GOLD and RUN domain. If we look into the evolutionary conservation of the FYCO1 protein sequence via CLUSTAL W, the scores show 81% alignment between Humans, dogs, and Cows and about 78% between humans and mice (Marchler‐Bauer et al., 2009). This high rate of conservation shows the significance of the protein and its functional implications at a large scale.

The correlation of autophagy and the role of *FYCO1* in the development of cataracts has been confirmed by Pankiv et al. (2010). His experiments with FYCO1 knockout mice suggested that the absence of FYCO1 protein resulted in the accumulation of perinuclear autophagosomes thus relating its key involvement in autophagy involves the formation of double membranous autophagosomes which engulf the damaged cellular components and direct them to lysosomes for further recycling thus maintaining the lens transparency (Yang & Klionsky, 2010). The absence of the Znf domain may lead to failure of this transportation and accumulation of vesicles. Autophagy has been one of the most extensively under investigation pathways since the 19th century but its involvement remains still unclear and controversial. However, it has been widely shown that autophagy plays an important role as intracellular quality control, but its implications in the development of CC and age‐related cataract is still a lot more to be worked out (Morishita & Mizushima, 2016).

## 5 CONCLUSIONS

In summary, we report here a novel splice site deletion (NM_024513.3: c.3151‐29_3151‐7del) in the exon 10 of *FYCO1* gene, resulting in congenital cataract in family 1. As a result of this mutation FYCO1 protein fails to adapt its necessary confirmation needed to perform its role in one of the key pathway autophagy for maintaining lens transparency. In addition, we also emphasize the presence of a higher frequency of mutations in the *FYCO1* gene in the cases reported for congenital cataract with a Pakistani origin thus increasing an overall frequency to 20% (5/25) which was previously 15% (Iqbal et al., 2020) and 13.8% (Chen et al., 2017). These findings propose considering the *FYCO1* mutation spectrum to be included in genetic testing platform particularly for cataract cases with Pakistani origin. Also, we propose there is a need for more functional and domain analysis studies for this protein and its implications in autophagy and lens development to help out in therapeutic applications.

## ACKNOWLEDGMENT

The authors thank the affected patients and their parents for their participation in the study. The authors also acknowledge the Department of Clinical Genetics, Academic Medical Centre, Amsterdam, Netherlands and Higher Education Commission, Pakistan and its International Research Support Initiative Program for providing this Research study opportunity.

## AUTHOR CONTRIBUTIONS

R.S.S., S.M., and S.I. conceived and designed the experiments; R.S.S. and S.M. performed the experiments; R.S.S., N.M.A., M.A.U.K., A.H., and S.M. performed results and bioinformatics analysis; S.N.S. and M.I.K. recruited patients, collected clinical data, and samples; S.N.S., M.I.K., S.M., and S.I. contributed reagents/materials/analysis tools; R.S.S., S.I., N.M.A., and S.M. wrote the manuscript. All authors have read and approved the final manuscript.

## Funding information

This research did not receive any specific grant from funding agencies in the public, commercial, or not‐for‐profit sectors.

## CONFLICT OF INTEREST

There is no conflict of interest as declared by the authors.

## ETHICAL COMPLIANCE

The approval of the study is done by Ethical Committee of the University of the Punjab (Lahore, Pakistan) and Institutional Review Board of the Al‐Shifa Eye Trust Hospital (Rawalpindi, Pakistan), and adheres to the tenets of the Declaration of Helsinki. As a prerequisite, appropriate written informed consents were signed and obtained from the participants or parents for the current study.

## Data Availability

The data that support the findings of this study are available from the first author upon reasonable request.

## Endnote

^1^
RefSeq (NM_024513.3) has an updated version available as (NM_024513.4)
